# Relaxation Dynamics in Polyethylene Glycol/Modified Hydrotalcite Nanocomposites

**DOI:** 10.3390/polym10111182

**Published:** 2018-10-24

**Authors:** Rossella Arrigo, Diego Antonioli, Massimo Lazzari, Valentina Gianotti, Michele Laus, Laura Montanaro, Giulio Malucelli

**Affiliations:** 1 Department of Applied Science and Technology and Local INSTM Unit, Politecnico di Torino, Viale T. Michel 5, 15121 Alessandria, Italy; rossella.arrigo@polito.it; 2 Department of Science and Technological Innovation, Università del Piemonte Orientale “A. Avogadro”, Viale T. Michel 11, 15121 Alessandria, Italy; diego.antonioli@uniupo.it (D.A.); valentina.gianotti@uniupo.it (V.G.); michele.laus@uniupo.it (M.L.); 3 Departamento de Química Física, Facultade de Química, and Centro Singular de Investigación en Química Biolóxica e Materiais Moleculares (CIQUS), Universidade de Santiago de Compostela, 15782 Santiago de Compostela, Spain; massimo.lazzari@usc.es; 4 Department of Applied Science and Technology and Local INSTM Unit, Politecnico di Torino, C.so Duca degli Abruzzi 24, 10129 Torino, Italy; laura.montanaro@polito.it

**Keywords:** rheological behavior, polyethylene glycol, organo-modified hydrotalcite, grafting reactions, annealing

## Abstract

Polyethylene glycol-based nanocomposites containing an organo-modified hydrotalcite with loadings ranging from 0.5 to 5 wt.% were prepared by melt mixing performed just above the melting point of the polymer matrix. In these conditions, the dispersion of the nanofiller within the polymer matrix was quite homogeneous as revealed by TEM analyses. The effect of various thermal treatments and filler loadings was thoroughly investigated by means of rheological, morphological and gas chromatography-mass spectrometry, hyphenated to thermogravimetry analysis tests. Unfilled polyethylene glycol exhibited a continuous decrease in complex viscosity upon heating. In contrast, the complex viscosity of nanocomposites containing nanofiller loadings higher than 1 wt.% showed first a decrease, followed by an increase in the complex viscosity as the temperature increases, exhibiting a minimum between 130 and 140 °C. Annealing at 180 °C for different times further increased the viscosity of the system. This unusual behavior was explained by the occurrence of grafting reactions between the –OH terminal groups of the polyethylene glycol chains and the hydroxyl groups of the organo-modified filler, thus remarkably affecting the relaxation dynamics of the system.

## 1. Introduction

Polymer-based nanocomposites have attracted a growing attention in the last few years, as the introduction of solid nanoparticles into a soft polymeric matrix allows for multi-functional materials with tailored and controlled properties, potentially suitable for advanced applications [1,2,3]. Compared to traditional composites, nanocomposites are characterized by a significantly increased interfacial area between the embedded nanoparticles and polymer matrices [4,5]. However, the full potential of this large interface can be effectively exploited only when the polymer-nanoparticle interactions are strong enough to overcome the intrinsic tendency of nanoparticles to self-aggregate [6]. Therefore, the dispersion and distribution of the incorporated nanoparticles, as well as the extent of interaction between the two components, is crucial to control the final properties of the resulting nanocomposite [7,8]. In addition, the polymer/nanofiller and nanofiller/nanofiller interactions remarkably affect the linear and non-linear rheological behavior of these materials [9,10]. In general, the viscoelastic behavior of polymer-based materials impacts on both the processing technology and conditions [11] and on the final properties [12] of the products. In addition, the rheological response of polymeric nanocomposites reflects their intimate microstructure, offering the possibility to gain a fundamental understanding of their structure-property relationships [12,13]. In this context, dynamic oscillatory measurements performed at low frequencies are capable of revealing fundamental information as far as the microstructural evolution of nanofilled polymers is concerned, being very sensitive to changes in nanocomposite internal structure and to the relaxation dynamics of polymer chains [14,15]. Furthermore, the low-frequency rheological behavior is strictly related to the interactions occurring within the nanostructured polymer-based materials and gives insight into the organization of the nanofillers within the host matrix and their arrangements in complex architectures [16]. In fact, when the formation of a percolated network of nanoparticles occurs, the motion of long polymer chain segments is restricted, thus resulting in a change in the relaxation spectrum of the material [17,18]. Usually, for polymer-based systems with uniform dispersion of nanofillers, a transition from liquid-like to solid-like rheological behavior is observed at relatively low content: this finding is clearly recognizable as the Newtonian behavior disappears, together with a divergence in complex viscosity [19,20], and a flattening in storage and loss moduli at low frequencies [21,22]. From another point of view, the temperature dependence of the rheological behavior of polymer melts is well known [21], including its effect on the microstructure [23]. Conversely, few works report on the influence of the temperature on the rheological behavior of polymer-based nanocomposites.

Due to the large number of commercially available grades with well characterized molar mass and narrow molar mass distribution, as well as their favorable processing characteristics, polyethylene oxides are ideal matrices [24] for investigating the rheological behavior of the corresponding composites. Kelarakis et al. [25] studied the rheological behavior of polyethylene oxide/clay nanocomposites as a function of their temperature, observing a remarkable increase in the melt viscosity as temperature increased and a pronounced solid-like character at high temperatures. This unusual behavior was attributed to the internal fluidity of the system, which allowed a reorganization of the polymer chains, facilitating the formation of a percolation network at high temperatures. Conversely, the thermoreversible physical gelation observed in the polyethylene oxide/carbon black system at temperatures far above the matrix melting point was ascribed to particle clustering phenomena, due to electrostatic particle-particle interactions [26].

In this context, we have recently studied the effect of boehmites and hydrotalcites on the thermal and rheological behavior of a polyethylene glycol (PEG) matrix [27]. Unlike boehmites, a homogeneous dispersion of nanoparticles within the polymer matrix was observed in hydrotalcites containing nanocomposites, thus affecting the rheological behavior of PEG. The presence of well dispersed hydrotalcites causes a slowdown in the relaxation dynamics of polymer chains and a consequent transition from liquid-like to solid-like behavior. Pursuing this research, the present work aims at assessing the effect of thermal treatments and filler loadings on the temperature dependence of the rheological behavior of PEG-based nanocomposites prepared by melt mixing and containing organo-modified hydrotalcite with loadings ranging from 0.5 to 5 wt.%. It can be anticipated that aside from some clustering phenomena, the experimental results clearly show the occurrence of grafting reactions between the nanofiller and the hydroxyl end-groups of PEG, which remarkably affect the relaxation dynamics of polymer chains.

## 2. Materials and Methods

### 2.1. Materials

Polyethylene glycol (PEG), purchased from Sigma-Aldrich (Saint Louis, MO, USA), was used as polymer matrix. The sample had an average molar mass ($\bar{M}$_n_) of 20,000 g/mol, with a polydispersity index of 1.1 and melting temperature of 65 °C.

A Magnesium–Aluminum Stearate Hydrotalcite (hereafter coded as LDHs) from Prolabin (Perugia, Italy) containing about 55 wt.% Al–Mg stearates as organo-modifiers was used as the nanofiller; main characteristics: Al_2_O_3_:MgO ratio = 37:63, surface area = 16 m^2^/g, loose bulk density = 150 g/L, powder particle size = 6 μm, crystallite size—XRD peak (003) = 100 nm.

### 2.2. Nanocomposite Preparation

The nanofiller powders were embedded within PEG by melt mixing, using a Brabender Plastograph mixer operating at 65 °C and 100 rpm for 3 min. These conditions were optimized in order to avoid any degradation phenomena of the polymer matrix [27]. The nanofiller loading was 0.5, 1, 1.5, 2.5, 3.5 and 5 wt.%.

Specimens for the rheological characterization were obtained by compression molding using a laboratory press (Collin Teach Line 200T, Ebersberg, Germany) at 65 °C for 2 min with a pressure of 100 bar. The unfilled PEG was subjected to the same processing.

### 2.3. Characterization

Rheological measurements were performed using an ARES (TA Instrument, New Castle, DE, USA) strain-controlled rheometer in parallel plate geometry (plate diameter: 25 mm), under a nitrogen atmosphere to avoid oxidative degradation.

The complex viscosity and storage and loss moduli were measured performing frequency scans from 10^−1^ to 10^2^ rad/s, at different temperatures. The strain amplitude was fixed at γ = 10%, which is low enough to be in the linear viscoelastic regime, as probed by strain sweep measurements carried out on all the prepared nanocomposites, but, at the same time, providing sufficient torque for rheological measurements. Temperature sweep tests were carried out from 65 to 180 °C at 2 °C/min, *ω* = 0.1 rad/s, γ = 10%. Before starting the rheological tests, each sample was put in between the rheometer plates and conditioned at 65 °C for 10 min, thus ensuring its complete melting. The typical gap between the plates imposed during the rheological analyses was 1 mm.

Transmission electron microscope (TEM) studies were performed on a JEOL JEM-1011 (Freising, Germany) working with an accelerating voltage of 100 kV. Ultrathin sections (nominal thickness of 80 nm) of PEG-based systems were cut at −40 °C using a Leica UC6 microtome (Wien, Austria) with an EM-FCS cryo kit equipped with a diamond knife, and collected onto formvar-coated copper grids.

Extraction tests were performed on all the nanocomposites. More specifically, samples were treated with THF in static mode, putting in contact 1 mg of sample with 10 mL of THF. Then, the remaining solid was separated from the solvent by centrifugation.

Gas chromatography-mass spectrometry hyphenated to thermogravimetry analyses were performed by a Mettler-Toledo TGA/SDTA 851e thermobalance (Columbus, OH, USA) and a FINNIGAN TRACE GC-ULTRA and TRACE DSQ gas chromatography-mass spectrometry system (Waltham, MA, USA). A scanning rate of 10 °C/min from room temperature to 1100 °C and a steady flow of He were employed in the TGA stage. The evolved gas from TGA was transferred to the GC-MS using a thermostatized interface [28] operating with the temperature of the transfer lines of 250 °C, the autoinjector temperature of 200 °C, a sampling frequency of 1.0 min^−1^. An injection loop with a volume of 2.5 mL was employed.

The chromatographic separation was carried out using a Phenomenex DB5-5ms capillary column (30 m, 0.25 mm i.d., 0.25 μm thickness). The injector temperature was set at 280 °C in splitless mode and He was used as carrier gas at a constant flow rate of 1.0 mL/min. The MS transfer line and the oven temperatures were set at 280 and 210 °C, respectively. The MS signal was acquired in EI+ mode with a 70.0 eV ionization energy. The ion source temperature was heated at 280 °C. The acquisition was performed both in full-scan mode in the 30–200 *m*/*z* range and in Single Ion Monitoring (SIM) mode by acquiring the signals of different *m*/*z* values, namely 18, 28, 30, 31, 44, 45, 59 and 73, corresponding to the fragments that evolve from PEG.

## 3. Results and Discussion

### 3.1. Effect of LDHs Loading

**Figure 1:**
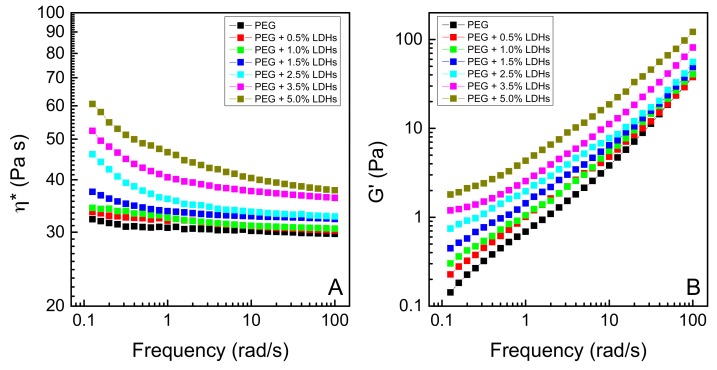
Complex viscosity η* (**A**) and Storage modulus G’ (**B**) at 65 °C as a function of frequency for unfilled PEG and all the investigated nanocomposites.

Oscillatory dynamic measurements were carried out within the material linear viscoelastic regime to evaluate the effect of different amounts of LDHs on the relaxation dynamics of PEG. Figure 1A,B shows the trends of complex viscosity (η*) and storage modulus (G’) at 65 °C as a function of frequency for unfilled PEG and all the investigated nanocomposites.

**Figure 2:**
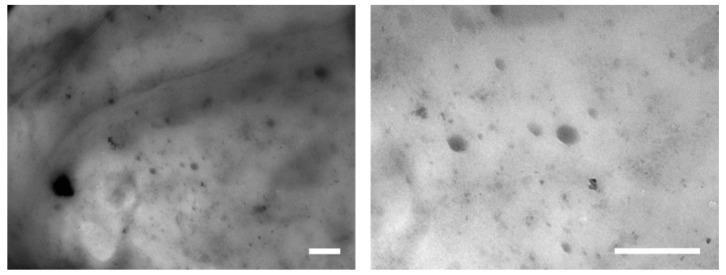
TEM pictures of PEG + 5.0% LDHs at two different magnifications (scale bar 500 nm).

The addition of LDHs leads to a viscosity increase in the whole frequency range, as compared to unfilled PEG. It is worth noticing that such an enhancement is progressively more pronounced as the content of LDHs increases. Furthermore, a remarkable modification of the trend of complex viscosity as a function of frequency is observed, especially in the low-frequency region, where the melt-state dynamics of large polymer segments are probed. In particular, the unfilled PEG, due to its low molecular weight, shows the typical Newtonian behavior, recognizable in the frequency independence of the complex viscosity over the entire investigated frequency range. In the nanocomposites, the presence of nanofillers causes the disappearance of the Newtonian plateau at low frequencies. This finding is a clear indication of the good dispersion of LDHs, regardless of their concentration [21]. To further support this hypothesis, TEM analyses were carried out on the different nanocomposite systems. As an example, Figure 2 shows two typical TEM pictures referring to the highest loaded nanocomposite at two different magnifications. It is worthy to note that LDHs particles are well dispersed within the polymer matrix and their size does not exceed about 400 nm with average dimensions in the range of 50–100 nm. Furthermore, the rheological response of nanocomposites containing loadings of LDHs higher than 1.5 wt.% suggests the formation of interconnected network-like structures. This hypothesis will be further investigated later.

Looking at the trends of G’ as a function of frequency depicted in Figure 1B, unfilled PEG exhibits the typical liquid-like terminal behavior (G’ *α* *ω*^2^) in the low frequency region, due to the full relaxation of the macromolecules. Upon incorporation of LDHs, the storage modulus of nanocomposites progressively increases in magnitude and the slope of the plot of G’ vs. *ω* at low frequencies gradually decreases as the loading of nanoparticles increases.

**Figure 3:**
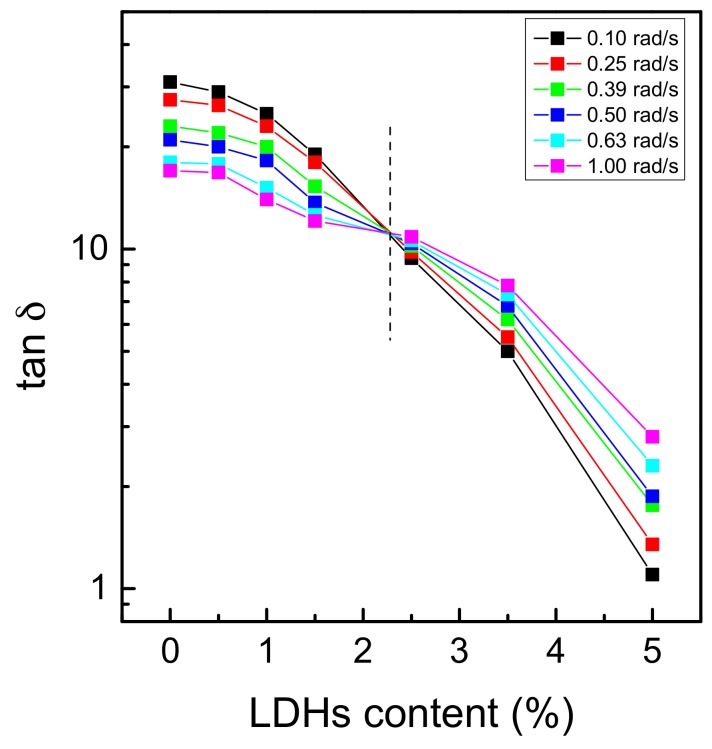
Loss tangent (tan δ) as a function of LDHs loading for PEG-based nanocomposites (T = 65 °C).

The observed deviation from the terminal behavior is indicative of a transition from liquid-like to solid-like rheological behavior, attributable to the fact that the long-range polymer chain relaxation is restrained by the presence of well-dispersed anisotropic nanofillers, arranged in a percolative network that spans throughout the matrix [29]. Usually, the filler concentration, at which G′ begins to exhibit non-terminal behavior, is referred as the percolation threshold, which marks the transition from liquid-like to solid-like rheological behavior [30]. It is well documented in literature that the formation and the evolution of a network of anisotropic nanoparticles in a polymer matrix can be considered as a kind of physical gelation [31,32,33]. In fact, a physical gel could be thought as a percolated three-dimensional network, in which the connectivity arises by physical interactions. In the case of a filled polymer at its rheological percolation, the macroscopic connectivity is provided by the different interactions taking place between the components (filler–filler, filler–polymer and polymer–polymer). Therefore, to analyze the rheological behavior of PEG/LDHs systems and to determine the percolation threshold, the well-established Winter-Chambon method was employed [34]. According to this approach, the percolation threshold (i.e., the gel point) can be determined from the frequency independence of loss tangent (tan δ = G′′/G′) in the low frequency region. Figure 3 shows a multi-frequency plot of tan δ as a function of nanoparticle loading. The general trend is a continuous decrease of tan δ with increasing LDHs content. The crossover point represents a frequency independent value of tan δ, and the LDHs amount, and the point at which this phenomenon occurs is the percolation threshold. Figure 3 clearly shows that for PEG/LDHs systems, at the investigated temperature, the percolation threshold is just below 2.5 wt.%.

### 3.2. Effect of Temperature

**Figure 4:**
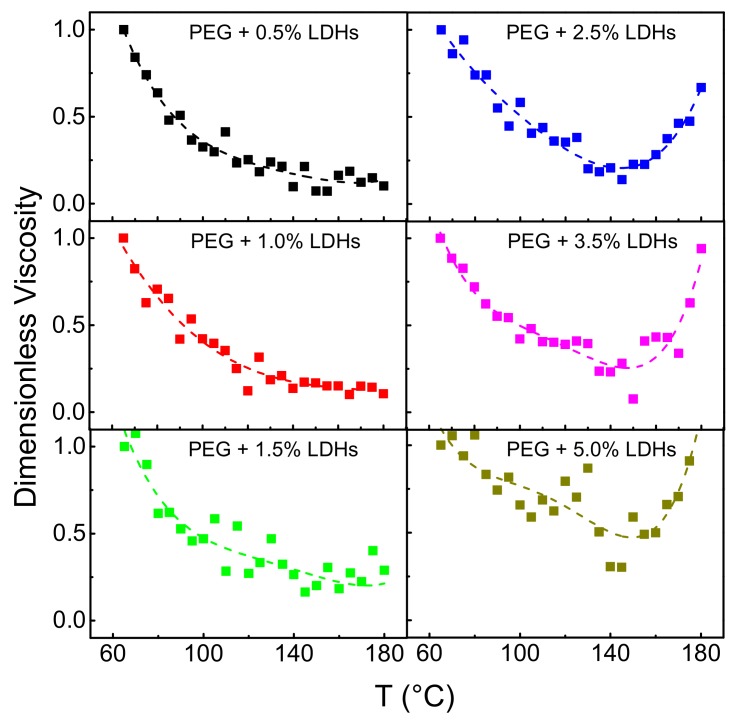
Dimensionless complex viscosity as a function of temperature for unfilled PEG and all investigated nanocomposites (*ω* = 0.1 rad/s).

Figure 4 shows the temperature dependence of complex viscosity of unfilled PEG and PEG-based nanocomposite melts, recorded during a single frequency heating scan performed at *ω* = 0.1 rad/s. Such a low value of frequency has been selected to approximate the zero shear behavior. Unfilled PEG and the nanocomposites containing low amounts of LDHs (up to 1.5 wt.%) exhibit a continuous decrease of η* upon heating, over the whole tested temperature interval. Conversely, the complex viscosity of nanocomposites containing higher loadings of nanofillers shows first a decrease upon heating, followed by an unexpected increase, exhibiting a minimum at 130–140 °C.

**Figure 5:**
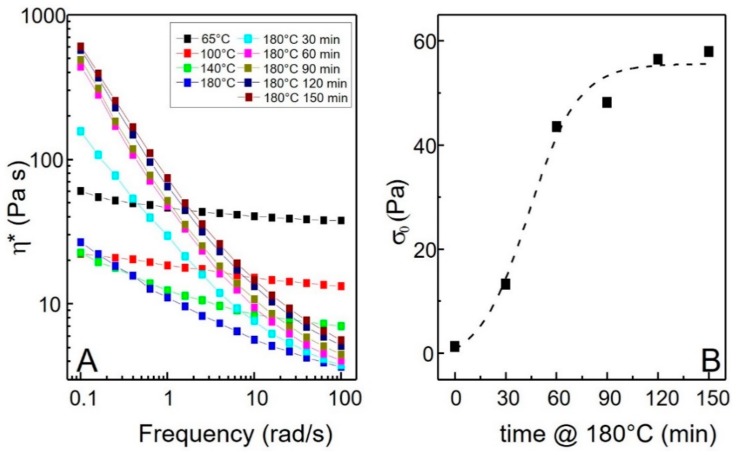
Trend of complex viscosity η* as a function of frequency for the PEG + 5% LDHs sample subjected to the different thermal treatments (**A**) and melt yield stress *σ*_0_ as a function of annealing time at 180 °C (**B**).

To gain a better understanding of this rheological behavior, isothermal frequency scans have been performed at different temperatures. Figure 5A shows the trends of complex viscosity for the nanocomposite containing 5 wt.% of LDHs. The increase in the temperature from 65 to 140 °C causes a decrease of the complex viscosity over the whole frequency range. At 180 °C, the values of the complex viscosity are lower than those recorded at 65 °C, but a more pronounced non-Newtonian behavior can be observed. When the annealing temperature is 180 °C, the complex viscosity increases with time, steeply at first, and then more gradually until a limiting behavior is reached, corresponding to 150 min of annealing time. Figure 5B reports the trend of melt yield stress *σ*_0_ as a function of annealing time at 180 °C. This parameter was calculated fitting the complex viscosity as a function of frequency using the Carreau-Yasuda model:

(1) $$\eta^{*}=\frac{\sigma_{0}}{\omega }+\eta_{0}{\left[1+{\left(\lambda \omega \right)}^{a}\right]}^{\left(n-1\right)/a}$$

where *σ*_0_ is the melt yield stress, *η*_0_ is the zero shear viscosity, *λ* is the relaxation time, *a* is the Yasuda parameter and n is the dimensionless power law index [10]. This model is divided in two parts; melt yield stress (low frequency) and zero-shear viscosity (high frequency). For this study, it is important to study the low frequency range to quantify the melt yield stress, and a sigmoidal trend of melt yield stress as a function of annealing time at 180 °C was found.

**Figure 6:**
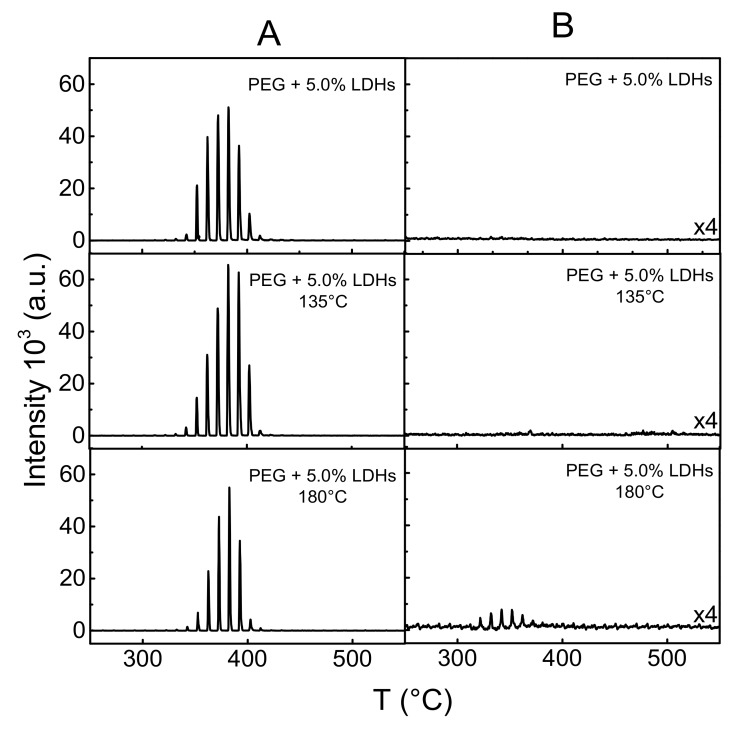
TGA-GC-MS chromatograms of the unextracted (**A**) and extracted (**B**) PEG + 5% LDHs sample annealed at 135 and 180 °C for 150 min.

These findings could be ascribed to the establishment of strong interactions between the PEG macromolecular chains and the dispersed LDHs nanoparticles. In fact, the hydroxyl termination of PEG macromolecules and the hydroxyl groups at the LDHs surface could interact during the annealing process, favoring the occurrence of some grafting reactions. To verify this hypothesis, the previously prepared samples, as well as the samples annealed at 135 and 180 °C for 150 min were analyzed by TGA-GC-MS. In addition, all these samples were further extracted with THF to remove the polyethylene glycol and the residue was analyzed. Figure 6 illustrates the TGA-GC-MS chromatograms of the samples, focusing on the *m*/*z* value of 73, typical for the loss of the ethylene glycol units. The chromatograms of the unextracted samples are very similar to each other and consist of a series of peaks relevant to ethylene glycol evolution due to the polyethylene glycol chain degradation. As the loss profiles are very similar for all the samples, it appears that the thermal treatments, even for relatively long time periods, do not induce degradation in the polyethylene glycol chains. Considering the solid residue after extraction, no trace of polyethylene glycol was observed in the previously prepared sample and in the sample annealed at 135 °C, thus clearly indicating that the washing procedure is highly effective in removing all the polyethylene glycol. In contrast, the TGA-GC-MS trace of the sample annealed at 180 °C reveals the presence of a small amount of PEG chains linked to the modified nanofiller.

Similarly, grafting reactions between a hydroxyl terminated polymer chain and hydroxyl groups at a silicon surface have been described in various systems, leading to the formation of thin films of the corresponding functional polymer onto the substrate [35,36]. In these systems, the grafting time for the full coverage of the surface depends on the molar mass of the functional polymer [37] and on the annealing temperature [38], which results in 300 h when the annealing temperature is 140 °C [35] but just some hours [36] when the grafting temperature is 180 °C. These results suggest that a grafting reaction between the hydroxyl terminal groups of the polyethylene glycol chains and the hydroxyl groups of the hydrotalcite occurs when the nanocomposites are annealed at temperatures higher than 135 °C.

## 4. Conclusions

Polyethylene glycol was exploited as a model matrix for investigating the relaxation dynamics occurring in its nanocomposites containing an organo-modified hydrotalcite at different loadings, when subjected to annealing processes performed at selected temperatures and times. In particular, unfilled polyethylene glycol and its nanocomposites containing low filler amounts (i.e., up to 1.5 wt.%) exhibited a continuous decrease of complex viscosity upon heating, over the whole temperature interval. Conversely, the nanocomposites containing higher nanofiller loadings showed an unusual increase in the complex viscosity in the terminal flow region. Gas chromatography-mass spectrometry hyphenated to thermogravimetric analyses, performed on the nanocomposite solid residues after extraction of the polyethylene glycol matrix in THF, revealed the occurrence of grafting reactions between the –OH terminal groups of the polyethylene glycol chains and the hydroxyl groups of the organo-modified filler. These findings suggest that the inherent reactivity between fillers and functional matrices can be exploited by thermal treatments, thus substantially influencing the rheology and melt processing behavior of a large variety of composites, including those based on poly(lactic acid), poly(lactic-co-glycolic acids) and those with terminal hydroxyl groups. The proposed nanocomposite systems may deserve further investigation as thermal energy storage materials with improved properties due to the interactions taking place between their two components.
